# Live cell imaging of *Plasmodiophora brassicae*—host plant interactions based on a two‐step axenic culture system

**DOI:** 10.1002/mbo3.765

**Published:** 2018-11-14

**Authors:** Jiangying Tu, James Bush, Peta Bonham‐Smith, Yangdou Wei

**Affiliations:** ^1^ Department of Biology University of Saskatchewan Saskatoon Canada

**Keywords:** *Arabidopsis*, axenic culture, calcofluor white, canola, DAPI, Nile red, *Plasmodiophora brassicae*, primary plasmodia, resting spores

## Abstract

*Plasmodiophora brassicae*, a parasitic protist, induces club‐shaped tumor‐like growth of host Brassicas roots and hypocotyls after infection. Due to its soil‐borne nature and intracellular, biotrophic parasitism the infection biology and early pathogenesis remains in doubt. In this study, we have established a new protocol, based on a two‐step axenic culture of *P. brassicae* with its host tissues, for easy and *in planta* observation of cellular interactions between *P. brassicae* and host plants: first, coculture of *P. brassicae* with infected canola root tissues, on growth‐medium plates, enables the propagation of *P. brassicae* that serves as pure inoculum for pathogenicity assays, and second, the pure inoculum is subsequently used for pathogenicity tests on both canola and *Arabidopsis* seedlings grown on medium plates in Petri dishes. During the first axenic culture, we established a staining protocol by which the pathogen was fluorescently labeled with Nile red and calcofluor white, thus allowing *in planta* observation of pathogen development. In the pathogenicity assays, our results showed that axenic cultures of *P. brassicae*, in calli, remains fully virulent and completes its life cycle in both canola and *Arabidopsis* roots grown in Petri dishes. Combining visualization of fluorescent probe‐labeled *P. brassicae* structures with fluorescent protein tagging of *Arabidopsis* cellular components, further revealed dynamic responses of host cells at the early stages of *P. brassicae* infection. Thus, established protocols for *in planta* detection of *P. brassicae* structures and the live cell imaging of *P. brassicae*—*Arabidopsis* interactions provide a novel strategy for improving our detailed knowledge of *P. brassicae* infection in host tissues.

## 1 INTRODUCTION

Clubroot, caused by the soil‐borne obligate intracellular parasite *Plasmodiophora brassicae*, is a serious, worldwide disease of the Brassicaceae. Infection by *P. brassicae* and subsequent disease progression result in a transformation of the host root, at site of infection, into a tumorous tissue, often restricting the uptake of water and nutrients into host plants. The aboveground tissues of a susceptible host crop become stunted, turn yellow, and are often pre‐ripening, resulting in significant yield and quality losses, accounting for a 10%–15% yield reduction on a global scale (Dixon, 2009). *Plasmodiophora brassicae* has three distinct stages to its life cycle: survival in soil as resting spores, primary infection of root hairs and epidermal cells where primary plasmodia, zoosporangia, and secondary zoospores are formed, and secondary infection resulting from released secondary zoospores that develop into multinucleate secondary plasmodia in the cortex and stele, resulting in cell hypertrophy and hyperplasy of infected roots (Kageyama & Asano, 2009). Finally, cleavage of secondary plasmodia produces numerous resting spores that are released into the soil, through infection tissue decay, where they can survive up to 20 years (Kageyama & Asano, 2009). *Plasmodiophora brassicae* displays a complex intracellular infection process involving various developmental stages during pathogenesis. Apart from resting spores released into the soil, the majority of *P. brassicae* life stages occur obligately inside distinct host tissues. Although much effort has been expended to understand the infection process, the intracellular, biotrophic life history of *P. brassicae* renders significant challenges to elucidating morphological and cellular characteristics of *P. brassicae* development in host tissues during clubroot disease initiation and development.

Many studies on host plant–*P. brassicae* interactions have been performed under artificial inoculation conditions in growth chambers and greenhouses, that is, flooding potting mix with resting spore suspensions (Sharma, Gossen, & McDonald, 2011a), growing a host plant in slurries of resting spores (Hwang et al., 2011), and using sand‐liquid culture (Agarwal, Kaul, Faggian, & Cahill, 2009; Deora, Gossen, & McDonald, 2013). However, observations of the infection process, especially during the early stages of infection, have proven difficult due to adherent soil and sand particles not easily removed by washing, subsequent root hair or root surface damage during root sample preparation and the presence of soil‐borne bacteria, fungi and oomycetes on and/or inside host tissues, increasing the complexity of this system for elucidating the *P. brassicae* life cycle.

In addition to the inoculation in soil and sand, several dual culture systems such as callus culture (Takahashi et al., 2001), hairy root culture induced by *Agrobacterium* (Asano, Kageyama, & Hyakumachi, 2000; Asano, Kodama, & Kageyama, 2006), cell suspension culture (Asano & Kageyama, 2006), and adventitious root culture (Takahashi et al., 2006) have been developed for maintaining an axenic culture of *P. brassicae* within infected host tissues. Many of these studies have used *Brassica napus* or *Brassica rapa* as host species and provided adequate systems for studying *P. brassicae*–host cell interactions avoiding the influence of other microbe contaminants.

Various histological methods have been used to study *P. brassicae*–host plant interactions (Schuller & Ludwig‐Müller, 2016), but routine techniques for selective labeling of pathogen structures have not been achieved. As we often see in reported studies on the cytology of clubroot, there is a striking resemblance between the primary plasmodium and the zoosporangium (sporulating plasmodium) of *P. brassicae* and host cellular compartments. In some instances, the appropriate identification of pathogen structures, using histological stains, has been rather ambiguous, especially during primary infection (Donald & Porter, 2004; Fei et al., 2016; Hwang et al., 2011; Sharma, Gossen, & Mcdonald, 2011b; Takahashi et al., 2006). Due to its obligate biotrophic nature, *P. brassicae* is not amenable for transgenic research, thus impeding the availability of fluorescent protein‐tagged strains for pathogen visualization during the infection process. Therefore, there is an urgent need to develop a methodology that will enable the *in planta* detection and observation of *P. brassicae*.

Abundant electron‐dense lipid droplets have been identified in *P. brassicae* plasmodia and resting spores (Deora et al., 2013; Williams & McNabola, 1967). The presence of enriched lipid droplets within pathogen structures allows for the pathogen to be distinguished from surrounding host cells of infected root tissues and may therefore serve as targets for histological staining. Lipid droplets have a core of neutral lipids, mainly triacylglycerides and sterol esters. In many studies, 9‐diethylamino‐5*H*‐benzo[a]phenoxazine‐5‐one (Nile red), a hydrophobic and metachromatic dye with poor solubility and fluorescence in water (Rumin et al., 2015), has been reported as a general lipid dye for both polar and nonpolar lipids using different excitation and emission wavelength ranges (Diaz, Melis, Batetta, Angius, & Falchi, 2008) in a wide range of biological systems (Guzmán, Jara, Duarte, & Presmanes, 2010; Kimura, Yamaoka, & Kamisaka, 2004; Kopischke et al., 2013; Miquel et al., 2014; Rumin et al., 2015). Detection of nonpolar, neutral lipids by Nile red staining is coupled with a short excitation wavelength such as EX/EM wavelengths of 488/550–650 nm, whereas detection of polar lipids can be achieved by Nile red staining using a long excitation wavelength of 530–560 nm (Guzmán et al., 2010; Li, Sun, Hicks, & Raikhel, 2015; Rumin et al., 2015). Nevertheless, Nile red staining for intracellular lipid droplets for identifying *P. brassicae* structures within clubroot tissues warrants further investigation and potential procedure optimization.

In this study, we have developed a two‐step dual culture protocol to obtain axenic cultures of *P. brassicae* from canola galls and to conduct pathogenicity assays on canola and *Arabidopsis* seedlings grown on medium plates in Petri dishes using pure inoculum from the axenic cultures. In addition, we have established and optimized live cell imaging techniques, by specific labeling of intracellular lipid droplets, the nucleus, and cell walls of the pathogen, that enable the identification of initial stages of the *P. brassicae* infection cycle. Combining visualization of fluorescent probe‐labeled *P. brassicae* structures and fluorescent protein tagging of *Arabidopsis* cellular components further revealed dynamic responses of host cells to *P. brassicae* infection. The live cell imaging based on an axenic culture system has proved to be a valuable tool for investigating the cellular and molecular mechanisms underlying *P. brassicae*–host interactions.

## 2 EXPERIMENTAL PROCEDURES

### 2.1 Pathogen inoculum

Canola, *B. napus* cv. “Westar,” was used to maintain the pathogen *P. brassicae* Woron. *P. brassicae* inoculum used in this study may represent a mixture of pathotypes, but pathotype 3 would certainly predominate (Peng et al., 2011). To prepare resting spores, 5 g of air‐dried clubroot galls were soaked in 200 ml distilled water for 2 hr to soften the tissue before grinding in a mortar and pestle. After filtering through seven layers of cheesecloth, the spore concentration was estimated using a hemocytometer and adjusted to 5.0 × 10^7 ^spores/ml. Canola seeds were sown in a soilless potting mix (Sunshine Mix #3/LG 3; Sun Gro Horticulture Canada Ltd., Canada) and plants were grown under 22°C, a 16 hr/8 hr light/dark photoperiod in a growth chamber (Conviron, USA). Five‐day‐old seedlings of canola were inoculated by pipetting 5 ml of resting spore suspension (5.0 × 10^7 ^spores/ml) around the base of the seedlings shoots, where it meets the soil.

### 2.2 Establishment of callus culture

The callus culture system was established according to Bulman et al. (2011) with minor modifications. Canola clubroot galls were harvested 5 weeks after inoculation. After washing thoroughly in tap water, galls were surface‐sterilized with 70% ethanol for 1 min, 30% bleach with 0.05% tween (~1.4% sodium hypochlorite) for 20 min, followed by three washes in sterile distilled water. After washing, galls were cut into sections between 2 and 3 cm in length, which were sterilized with 70% ethanol for 1 min, 2% Chloramine‐T (Sigma‐Aldrich, Oakville, Canada) for 20 min, followed by three washes in sterile distilled water. After this second sterilization, the sections were cut into ~0.5 cm segments and were placed on a medium consisting of Gamborg B5 basal medium (Sigma‐Aldrich, Oakville, Canada) plus 3% sucrose, 300 mg/L timentin (Gold Biotechnology, St. Louis, US), and 1.5% agar. Lateral root segments of non‐inoculated plants were used as control, because mature main roots were inflexible and too difficult to cut into small segments.

After 2 week incubation in the dark at 23°C, gall segments showing callus growth on the cut edges were transferred to fresh medium minus timentin. At the same time, a thin section was removed from each cultured segment, divided into two pieces, with one piece placed into Luria–Bertani (LB) liquid medium and one onto Potato Dextrose Agar (PDA) medium to confirm that the gall/callus segment was free of any endophytic bacteria and fungi. Each medium was observed for 3 days to confirm no bacterial or fungal growth. Endophytic microbe‐free calli were used to inoculate host seedlings.

### 2.3 Pathogenicity assay of canola and *Arabidopsis* seedlings grown in Petri dishes

Pathogenicity testing of canola and *Arabidopsis* seedlings grown on medium plates in Petri dishes, used canola cultivar Westar and *Arabidopsis thaliana* Col‐0 ecotype. Westar seeds, surface‐sterilized for 1 min in 70% ethanol, followed by 20 min in 30% bleach and three washes in sterile water, were grown in square Petri dishes on half‐strength Murashige and Skoog (MS) medium in a growth chamber at 23°C with a 16 hr/8 hr, light/dark photoperiod (light intensity 120 µmol/ms). Approximately 10 canola seeds or 15–20 *Arabidopsis* seeds were germinated and grown on MS plate in Petri dish. After 4 days, each seedling was inoculated with 400 µl of 5.0 × 10^7 ^spores/ml resting spore suspension, freshly prepared from callus, by pipetting onto the roots. Petri dishes were sealed thoroughly with Parafilm to confine the internal environment. The inoculation procedure in *Arabidopsis* was similar with one difference: The time for bleach sterilization was reduced to 5 min. The infection process was observed under transmission light microscopy (AxioPlan; Zeiss). The pathogenicity tests have been repeated at least three times on both *Arabidopsis* and canola seedlings. *Arabidopsis* transgenic lines, expressing PMA4‐GFP (Nottingham Arabidopsis Stock Centre, stock number N799380 (Teh & Moore, 2007)) and AT4G19150‐GFP (Arabidopsis Biological Resource Centre, stock number CS84731 (Cutler, Ehrhardt, Griffitts, & Somerville, 2000)) were infected using the same inoculation procedure.

### 2.4 Live *in planta* staining of *Plasmodiophora brassicae*

During tissue culture, Nile red (Sigma‐Aldrich, Oakville, Canada) and calcofluor white (CFW, Sigma‐Aldrich, Oakville, Canada) double‐staining techniques were used to observe pathogen development in calli. Calli were incubated in a final solution of 1 µg/ml Nile red, diluted in sterile water from a 10 mg/ml stock, for 10 min in the dark. After a wash with sterile water, calli were incubated in two drops of CFW for 5 min in the dark following the supplier^'^s instruction. After a final wash, calli were mounted on glass slides for observations.

For live cell imaging of *P. brassicae*–*Arabidopsis* interactions, 4,6‐Diamidine‐2‐phenylindole dihydrochloride (DAPI, Sigma‐Aldrich, Oakville, Canada) staining of inoculated *Arabidopsis* roots was carried out in a working solution of 1 μg/ml DAPI, diluted from a 10 mg/ml stock in water, for 20 min in the dark, followed by a wash with sterile water and where appropriate, 1 µg/ml Nile red was added for 5 min. Images of stained roots were viewed and captured with a confocal laser‐scanning microscope (Zeiss Meta 510).

### 2.5 Confocal microscopy

To observe neutral lipids stained with Nile red an excitation wavelength of 488 nm, with emission recorded with a 585–615 nm filter set, was used. For polar lipids, an excitation wavelength of 543 nm was used, and emission recorded with a >650 nm filter set. For both CFW and DAPI stained samples, the excitation wavelength was 405 nm with emission measured between 420 and 480 nm. For imaging GFP, a 488 nm excitation wavelength was used with GFP fluorescence detected with a 505–530 nm band pass. All observations were obtained from at least three independent experiments. After acquisition, images were analyzed and processed with LSM Image Browser and Adobe Photoshop (Adobe Systems). Contrast and brightness levels were optimized.

## 3 RESULTS

### 3.1 Establishment of dual culture of calli harboring *Plasmodiophora brassicae*

**Figure 1:**
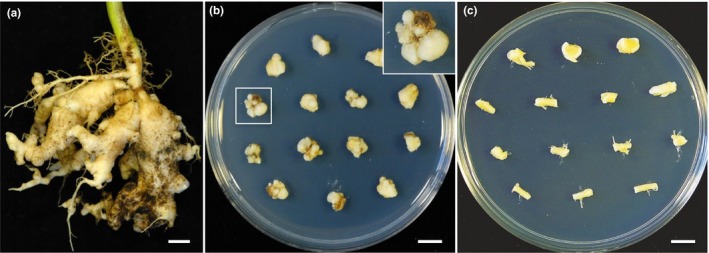
Culture of clubroot gall segments of *Plasmodiophora brassicae*‐infected canola roots on MS medium plates. (a) Canola roots infected with *P. brassicae* at 35 days post inoculation. (b) Calli formed on the surface of clubroot gall segments after 2 weeks culture on Gamborg B5 plates. Image in top corner is the enlarged view of the boxed area. (c) Segments of uninoculated canola roots cultured on Gamborg B5 plates for 2 weeks. Note that no callus formed on the surface of uninoculated canola roots. Scale bars: 1 cm

Tissue culture of clubroot gall segments of *P. brassicae*‐infected canola roots at 35 days post inoculation (dpi) was established on Gamborg B5 medium plates without the addition of any plant growth regulators. To prevent other soil‐borne microorganisms from confusing our observation of the *P. brassicae* life cycle in host *B. napus*, we established a modified Bulman dual culture system, taking care to remove all possible contaminating microorganisms (Bulman et al., 2011). Five days after incubation on B5 plates, even in the presence of 300 mg/L timentin, some of the surface‐sterilized segments of *B. napus* clubroot galls frequently displayed either bacterial or fungal contaminations. Subsequently, uncontaminated segments were transferred to fresh medium plates with 300 mg/L timentin. After further incubation for 2 weeks, it became apparent that these transferred gall segments were free of obvious microbial contamination, and outgrowth (callus‐like structures) of plant tissues/cells on the peripheral surface of the segments frequently appeared, whereas the non‐inoculated *B. napus* root tissue segments did not produce visible calli on B5 medium plates in the absence of plant growth regulators (Figure 1).

After 2 weeks incubation on B5 medium plates with antibiotics, uncontaminated calli were transferred to fresh B5 plates in the absence of antibiotics, and at the same time thin sections from these calli were transferred to nutrient‐rich liquid LB medium and solid PDA medium plates to double check whether calli were indeed free of contaminating bacteria and fungi. Calli free of contamination were used in further examinations.

### 3.2 Optimization of differential visualization of intracellular lipid droplets and membrane lipids stained with Nile red

**Figure 2:**
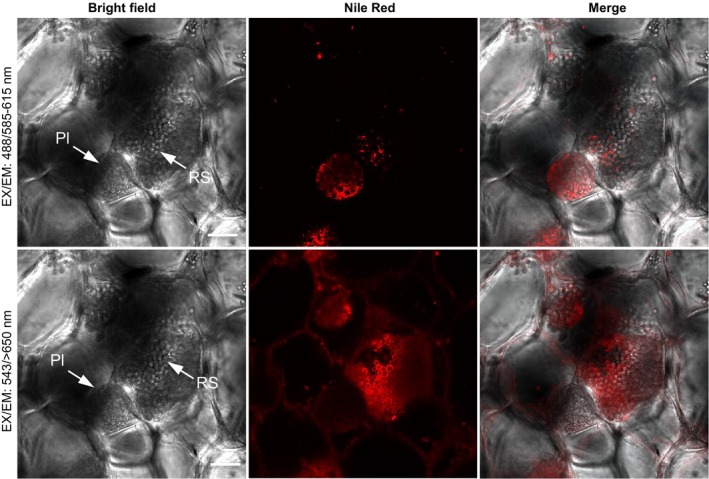
Confocal images of secondary plasmodia and resting spores in calli derived from *Plasmodiophora brassicae*‐infected canola galls. The same callus tissue was viewed under different confocal settings after staining with Nile red. Top panel: Intracellular lipid droplets within a secondary plasmodium were stained by Nile red and visualized by confocal microscopy with excitation and emission wavelengths 488/585–615 nm. Bottom panel: Plasma membrane (PM) of *P. brassicae* resting spores and PM of host cells were stained by Nile red and visualized with alternative excitation and emission wavelengths 543/>650 nm. Pl: secondary plasmodium; RS: resting spores. Scale bar: 20 μm

Previous studies had demonstrated that secondary plasmodia and resting spores of *P. brassicae* contain abundant intracellular lipid droplets (Deora et al., 2013; Williams & McNabola, 1967) and that the mature resting spores contain chitin in cell walls (Moxham & Buczacki, 1983; Schwelm et al., 2015). To facilitate the examination of structures and development of *P. brassicae* in *B. napus* callus tissues, we employed two fluorescence probes, Nile red and CFW, for detection of lipid droplets and chitin cell walls, respectively. Nile red, a lipophilic fluorescent marker, has been used previously to stain intracellular lipid droplets, abundant in secondary plasmodia and resting spores of *P. brassicae* (Deora et al., 2013; Williams & McNabola, 1967). CFW, selectively binding to β1–3 and β1–4 polysaccharides such as those found in cellulose and chitin, was used to label chitin in the cell walls of mature resting spores of *P. brassicae* (Moxham & Buczacki, 1983; Schwelm et al., 2015). To develop an applicable staining method for detection of intracellular *P. brassicae* in host tissues, the optimization of Nile red staining for cellular lipids was further investigated. Confocal microscopy showed that abundant intracellular lipid droplets in secondary plasmodia in callus cells infected by *P. brassicae* were readily observed by Nile red staining coupled with an excitation/emission (EX/EM) wavelength set of 488/585–615 nm (Figure 2, top panel). In contrast, the Nile red‐stained plasma membranes of host cells and cell membranes of resting spores were clearly visible with an EX/EM wavelength set of 543/>650 nm (Figure 2, bottom panel), but no signals for intracellular lipid droplets were detected within both secondary plasmodia and resting spores under this confocal setting (Figure 2, bottom panel). Furthermore, Nile red staining at an EX/EM wavelength set of 488/585–615 nm showed abundant intracellular lipid droplets within secondary plasmodia and developing resting spores of *P. brassicae*, but were barely detected in cellular compartments of *P. brassicae*‐infected host cells.

**Figure 3:**
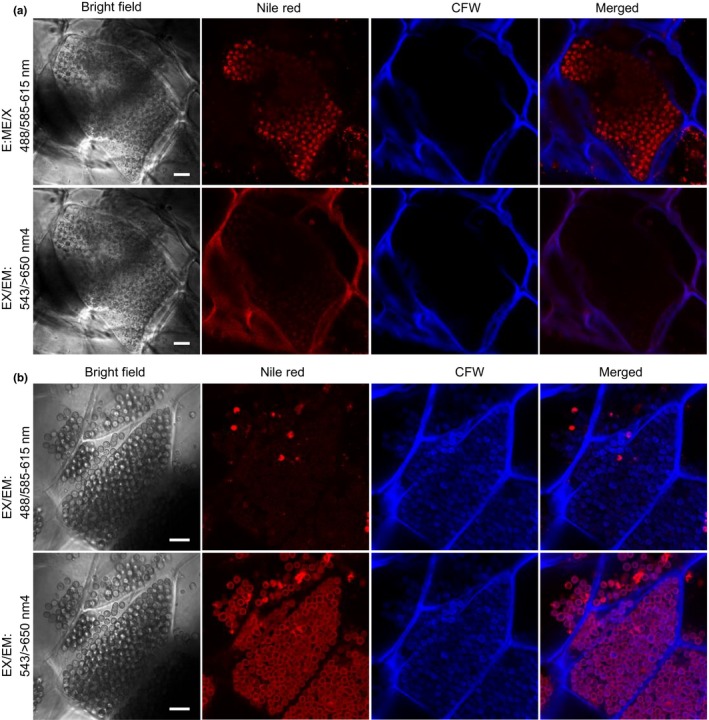
Confocal images of resting spore formation in callus cells generated from *Plasmodiophora brassicae*‐infected canola gall segments. (a) Immature resting spores in callus cells stained with Nile red and calcofluor white (CFW). Top panel: Fluorescent signals for Nile red‐labeled intracellular lipids of immature resting spores examined with EX/EM wavelengths 488/585–615 nm. Bottom panel: Fluorescent signals for Nile red staining preferentially labeled plasma membrane of infected callus cells rather than intracellular lipid droplets of immature resting spores examined with alternative EX/EM wavelengths 543/>650 nm. The host cell wall was also stained by CFW. Scale bars: 10 μm. (b) Mature resting spores in callus cells stained with Nile red and CFW. Top panel: No fluorescent signals were detected from intracellular lipid droplets of mature resting spores by Nile red staining using EX/EM wavelengths 488/585–615 nm. Bottom panel: Fluorescent signals were detected at the PM of mature resting spores using alternative EX/EM wavelengths 543/>650 nm. Cell walls of both mature resting spores and host cells were stained by CFW. Scale bars: 10 μm

When viewed after Nile red staining, with either wavelength set, intracellular lipid droplets were not detected in resting spores in infected host tissues (Figure 2), even though lipid droplets, as shown by transmission electron microscopy (TEM) imaging, were abundant in resting spores (Williams & McNabola, 1967 and Tu J, Wei Y, and Bonham‐Smith P. unpublished data). The inability of Nile red to stain lipid droplets in resting spores indicates that the cell wall deposition of resting spores may prevent the uptake of Nile red dye into intracellular compartments and subsequent lipid droplet labeling. To investigate whether cell wall deposition may interfere with Nile red uptake into resting spores, we introduced CFW to visualize the chitin cell wall of developing resting spores. Resting spores in callus tissues at different developmental stages were further examined under confocal microscopy after simultaneous labeling lipids with Nile red and cell wall chitin with CFW. Intracellular lipid droplets in immature resting spores, as revealed by negative staining with CFW (Figure 3a), were highly visible by Nile red with an EX/EM wavelength set of 488/585–615 nm, whereas no staining of intracellular lipid droplets was observed in mature resting spores with cell walls revealed by strong CFW staining (Figure 3b, top panel). Using the wavelength set 543/>650 nm, cell membranes of mature resting spores (Figure 3b, bottom panel), but not immature resting spores (Figure 3a, bottom panel), were strongly labeled by Nile red. The differential labeling of immature and mature resting spore membranes by Nile red (Figure 3a,b, bottom panels) suggests that changes in membrane lipid composition may occur during resting spore maturation.

In summary, intracellular lipid droplets of *P. brassicae* in host tissues are specifically stained by Nile red and optimally visualized under confocal microscopy at excitation of 488 nm and emission of 585–615 nm. Additionally, immature and mature resting spores of *P. brassicae* were clearly differentiated by CFW staining of cell wall chitin depositions during resting spore maturation. This staining by CFW confirmed the production of mature resting spores within infected callus cells, an indication of completion of the *P. brassicae* life cycle in the axenic dual culture system.

### 3.3 Pathogenicity of *Plasmodiophora brassicae* inoculum derived from axenic culture on host seedlings grown in Petri dishes

**Figure 4:**
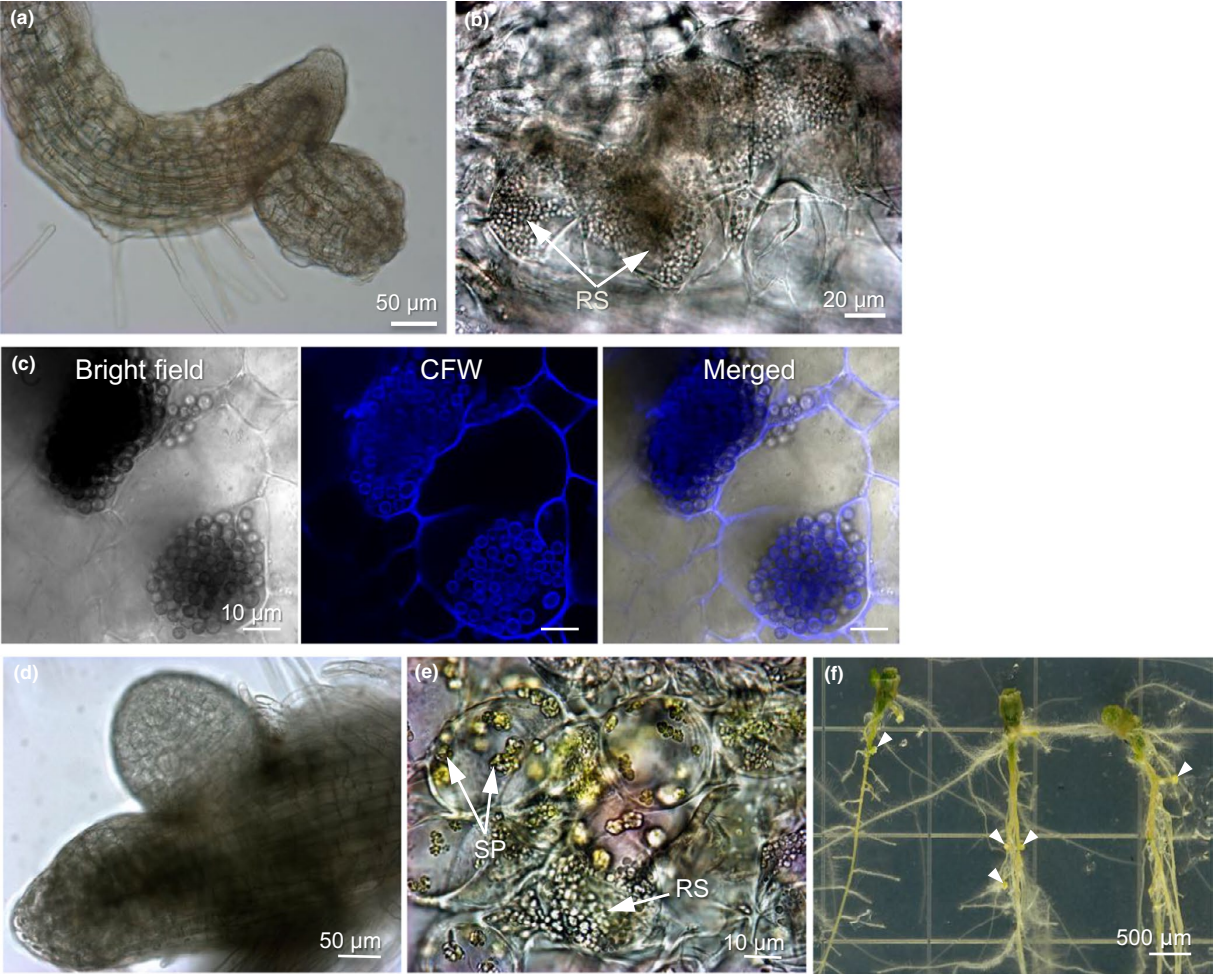
*Plasmodiophora brassicae* successfully proliferated and completed its life cycle in both *Arabidopsis* and canola roots grown in Petri dishes. (a) A small gall emerging from the epidermis of the elongation zone of *Arabidopsis* roots at 45 days post inoculation (dpi). (b) Microscopic view of a thin section of gall tissues showing resting spores formed in parenchyma cells of the infected gall on *Arabidopsis* roots at 60 dpi. (c) Formation of mature resting spores in infected galls of *Arabidopsis* roots. Cell walls of mature resting spores in infected galls at 60 dpi were stained by CFW (blue) and reviewed under confocal microscopy. (d) Microscopic view of a young gall emerging from the epidermis of the root elongation zone of canola root at 30 dpi. (e) Microscopic view of a thin section of gall tissue showing plasmodia and resting spores of *P. brassicae* at 30 dpi. SP: secondary plasmodia; RS: resting spores. (f) Development of visible clubroot galls on canola roots at 45 dpi. Small galls (arrowheads) appeared on the surface of both main and lateral roots

Resting spore suspensions of *P. brassicae* from calli were freshly prepared and used to inoculate both *Arabidopsis* and canola seedlings grown in Petri dishes, and proliferation of the pathogen within the infected roots was observed by microscopy. Approximately 6 weeks post inoculation, small galls emerged from epidermal cells in the elongation zone of *Arabidopsis* roots and abundant resting spores were observed within the parenchymal cells of these galls (Figure 4a,b). At 60 dpi, sections from these small galls were stained with CFW and examined under confocal microscopy. Resting spores stained with CFW for cell wall depositions were clearly visible (Figure 4c). Similarly, at 30 dpi with the resting spore suspension freshly prepared from callus tissues, small galls were observed under light microscopy, on the surface of the elongation zone of canola roots grown in Petri dishes (Figure 4d). At this stage, abundant secondary plasmodia and resting spores in the parenchymal cells of small galls were observed (Figure 4e). At 45 dpi (Figure 4f), galls along the surface of canola roots were clearly visible by eye, and 3–10 visible galls appeared on the root surface of each infected seedling. Taken together, our results indicate that inoculum derived from the axenic dual culture of canola calli exerts full virulence on root tissues of both *Arabidopsis* and canola hosts grown on medium plates in Petri dishes.

### 3.4 Live cell imaging of *Plasmodiophora brassicae*–*Arabidopsis* interactions during early infection stages

**Figure 5:**
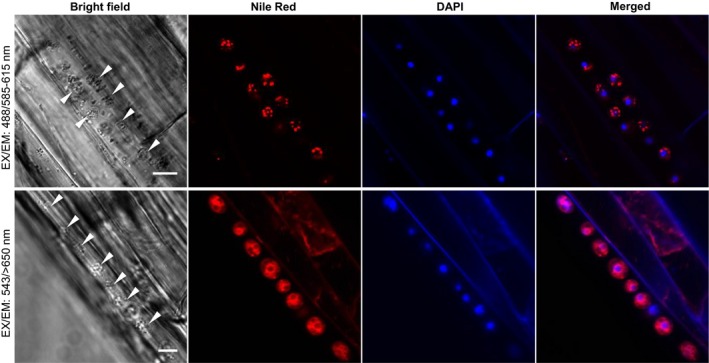
Confocal images of primary plasmodia in root epidermal cells of *Arabidopsis* seedlings grown on MS plates. *Arabidopsis* seedlings grown on MS plates for 7 days were inoculated with inoculum derived from the axenic culture of *Plasmodiophora brassicae* in canola root tissues. Infected root epidermal cells of *Arabidopsis* seedlings at 2 days post inoculation were stained with Nile red and DAPI and viewed under a confocal microscopy. Top panel: Intracellular lipid droplets and nuclei of primary plasmodia stained by Nile red and DAPI were visualized with EX/EM wavelengths 488/585–615 nm (red) and 405/420–480 nm (blue), respectively. Bottom panel: The endomembrane and nuclei of primary plasmodia stained by Nile red and DAPI were visualized with alternative wavelengths 543/>650 nm (red) and 405/420–480 nm (blue), respectively. Arrowheads indicate primary plasmodia in epidermal cells of the root elongation zoon. Scale bars: 10 μm

Having shown that *P. brassicae* derived from callus tissue retains full virulence with the production of mature resting spores in *Arabidopsis* root tissues grown on medium in Petri dishes, we wished to establish a live cell imaging system to study *P. brassicae*–*Arabidopsis* interactions and pathogenesis during early infection stages, that remain largely unknown in the infection cycle (Schuller & Ludwig‐Müller, 2016). With a high inoculation pressure (5 × 10^7^–10^8 ^spores/ml), inoculated *Arabidopsis* roots were shorter than control roots, and multiple primary plasmodia colonized epidermal cells of the root elongation zone could be observed at 2 dpi (Figure 5). The *P. brassicae* primary plasmodia were spherical and approximately 5 μm in diameter. To confirm these spherical structures as the pathogen rather than host cell organelles, infected *Arabidopsis* roots were double‐stained with Nile red and DAPI. Nile red‐stained intracellular lipid droplets were observed at 585–615 nm after excitation at 488 nm (Figure 5, top panel), whereas the endomembrane compartments of primary plasmodia were observed at >650 nm after excitation with 543 nm (Figure 5, bottom panel). The single nucleus of each primary plasmodium was fluorescently labeled blue with DAPI, indicating primary plasmodia were uninucleate up on initial formation after infection (Figure 5). Previously, as identified by TEM in an infected cabbage root hair, this stage had been referred to as uninucleate ameba (Aist & Williams, 1971). As a protist in the eukaryotic kingdom, primary plasmodia of *P. brassicae* at early infection stages in host cells contain complex cellular components including nuclei, lipid droplets, and an endomembrane system.

**Figure 6:**
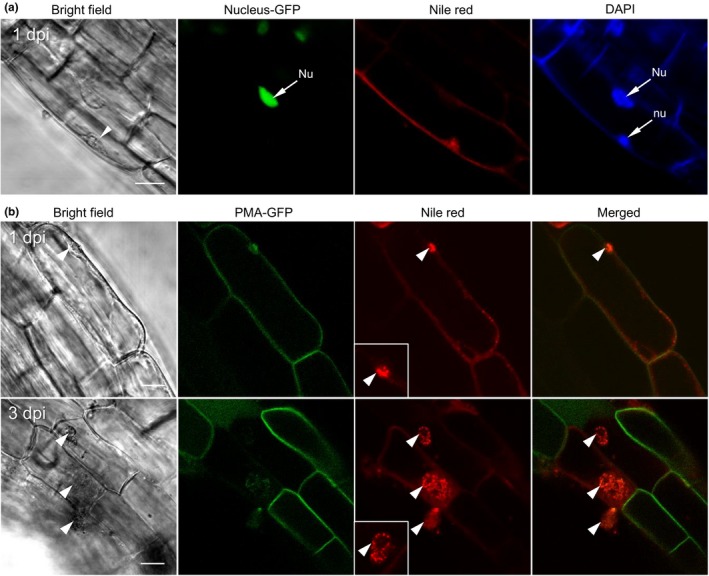
Live cell imaging of primary infection of *Plasmodiophora brassicae* in *Arabidopsis* roots. *Arabidopsis* seedlings expressing a 35S::AT4G19150‐GFP (CS84731) construct for labeling the nucleus (a) or a 35S::PMA4‐GFP (N799380) construct for labeling plasma membrane (b) were grown on MS plates for 7 days. Roots of *Arabidopsis* seedlings were inoculated with inoculum derived from the axenic culture of *P. brassicae* in canola root tissues. Infected *Arabidopsis* roots were stained with Nile red and/or DAPI and viewed under confocal microscopy. Inserts show images of single plasmodia with abundant lipid droplets. Arrowheads indicate primary plasmodia in epidermal cells of the root elongation zoon. Nu: host nucleus; nu: *P. brassicae* nucleus. Scale bars: 10 μm

Advantages of live cell imaging prompted us to examine the interaction of *P. brassicae* with the host cellular responses by using transgenic *Arabidopsis* plants expressing a GFP fusion of the nuclear protein, AT4G19150‐GFP and the plasma membrane (PM) protein, PMA4‐GFP, respectively. *Arabidopsis* seedlings grown on MS medium were inoculated with *P. brassicae* inoculum from the dual culture and then examined by confocal microscopy after staining with Nile red and DAPI. At 1 dpi, a root epidermal cell of the AT4G19150‐GFP‐expressing plant contained a primary plasmodium of *P. brassicae* that was located at the host cytoplasmic periphery and clearly labeled by Nile red and DAPI (Figure 6a). The infected epidermal cell displayed a nuclear GFP signal co‐localizing with the DAPI staining. Similarly, an epidermal cell of the PMA4‐GFP‐expressing plant with a primary plasmodium at the cytoplasmic periphery showed the green fluorescence of the PM marker (Figure 6b). These images confirmed that *Arabidopsis* cells hosting primary plasmodia at the initial infection stage maintained cellular integrity. At 3 dpi, intracellular primary plasmodia labeled by Nile red were observed in infected root epidermal cells of the PMA4‐GFP‐expressing plant. In these infected cells, the green fluorescence of the PM marker disappeared compared to adjacent epidermal cells where no pathogen was visible and the PMA4‐GFP signal was retained (Figure 6b). These observations suggest that either the PM has been specifically depleted of the PMA4‐GFP proteins or that it generally disintegrated when intracellular primary plasmodia were formed within the infected cells. In either case, whether this is actively triggered by the pathogen during pathogenesis or constitutes a defense response of the host cell remains to be elucidated.

## 4 DISCUSSION

The aim of this study was twofold: (a) to establish an axenic culture system for the production of pure inoculum of *P. brassicae*; and (b) to develop an *in planta* staining protocol for observation and detection of *P. brassicae* infection structures at the early stages of pathogenesis on medium plates using pure inoculum from the axenic culture.

In developing the axenic culture system, we followed the method developed by Bulman et al. (2011) with some modifications, in that we added a “callus cleaning” step to ensure that final calli were completely free of any microbial contaminations. Upon inoculation with resting spores of *P. brassicae* prepared from diseased root tissues, in addition to the *P. brassicae*, other pathogenic, saprophytic, and/or endophytic microorganisms are often associated with the infection in diseased root tissues, which can result in difficulties discerning *P. brassicae* structures within infected tissues and has often resulted in ambiguous identification of *P. brassicae* structures (Asano et al., 2000; Donald & Porter, 2004; Fei et al., 2016; Hwang et al., 2011; Sharma, Gossen, & Mcdonald, 2011b; Takahashi et al., 2006). In this study, pathogenicity tests on host *Arabidopsis* and canola plants grown on medium plates in Petri dishes, after inoculation with pure inoculum of *P. brassicae* from an axenic culture, are free of any microbial contamination, and provide an ideal pathosystem with a stable, controlled microenvironment, for the investigation of early interactions between *P. brassicae* and its host plants. The established system significantly facilitates a direct and rapid observation of clubroot disease development and the *in planta* observation of *P. brassicae* structures during the infection process. Using this technique, our studies revealed that epidermal cells of the hairless root elongation zone and root hairs close to this region are the most sensitive to an initial pathogen invasion and colonization. At 2 days post inoculation (dpi), epidermal cells of the root elongation zone were heavily colonized with uninucleate primary plasmodia (Figure 5). Moreover, at the later stages of disease development, the first galls always emerged from epidermal cells of the elongation zones of both *Arabidopsis* and canola roots (Figure 4). These observations are consistent with previous reports that several root hair deficient mutants of *Arabidopsis*, including the hairless mutants *rhd4‐1* and *axr1‐12*, are as susceptible to *P. brassicae* infection as wild‐type *Arabidopsis* plants (Siemens, Nagel, Ludwig‐Müller, & Sacristán, 2002). Rapid expansion of cells at the root elongation zone is coordinated by increased hydraulic pressure exerted from an osmolyte‐filled vacuole together with the controlled relaxation of cell walls, as a result of polysaccharide interlinks being broken (Verbelen, Cnodder, Le, Vissenberg, & Baluška, 2006). This process likely contributes to ease of initial penetration and subsequent colonization by *P. brassicae* of epidermal cells within root elongation zones. Whether initial infection and colonization of *P. brassicae* in epidermal cells of the root elongation zone occurs under natural conditions in the field requires further investigation.

The production of mature resting spores within small galls on the root surface of *P. brassicae*‐inoculated seedlings in Petri dishes indicates the completion of *P. brassicae* life cycle in the axenic culture system. However, under axenic culture conditions, gall formation was delayed and the size of galls was relatively small when compared to the disease development in which pathogenicity tests were conducted in soil conditions. We also observed that the level of the primary and secondary infection under the axenic culture system was much lower than that observed on soil‐grown plants at the same stages after inoculation with the same inoculum density. This discrepancy could be due to the difference of the microenvironment between culture media and soil, which may involve pH, exposure to light, nutrient availability, presence of other microorganisms, and plant growth. For example, at the same growth stages, *Arabidopsis* plants grown on medium plates are generally much smaller than plants grown in soil. Since *P. brassicae* is an obligate intracellular parasite, and its nutrients are being supplied almost exclusively from the infected host cells. Restricted growth of the host plant and its cells on the medium plates may provide the nutrient limitation that will affect the rapid, massive growth, and reproduction of *P. brassicae* in host tissues, thus reducing gall enlargement.

One of the key challenges in clubroot research is the lack of a reliable method of *in planta* detection of the *P. brassicae* infection structures inside plant cells. Unlike most plant microbial pathogens, *P. brassicae* is entirely intracellular, and during its infection cycle, several forms of developmental stages are present in various host cells/tissues. Clubroot research therefore requires dedicated tools for microscopic visualization of *P. brassicae*. Previous studies using TEM demonstrated that abundant intracellular lipid droplets were present in secondary plasmodia and resting spores of *P. brassicae* (Deora et al., 2013; Williams & McNabola, 1967; Williams & Yukawa, 1967). This key finding has led us to identify new histological probes for the observation of *P. brassicae* structures during the infection process. We have employed Nile red, which is cell permeable and binds to polar and nonpolar lipids in live cells, for labeling and visualization of intracellular lipid droplets of *P. brassicae* by confocal microscopy using excitation/emission wavelengths of 488 nm/585–615 nm. Our results are indeed consistent with past reports, demonstrating that neutral, nonpolar lipid droplets are abundant in secondary plasmodia and resting spores as well as in primary plasmodia of *P. brassicae* (Deora et al., 2013; Williams & McNabola, 1967; Williams & Yukawa, 1967). In contrast, lipid droplets were barely detected in cellular compartments of host *Arabidopsis* and canola cells of infected root tissues. Thus, Nile red staining of intracellular lipid droplets allowed us to specifically label *P. brassicae* within host cells/tissues.

To optimize *in planta* labeling and detection of *P. brassicae*, we further developed a double‐staining protocol using Nile red with DAPI for nuclear staining or CFW for cell wall staining of *P. brassicae*. DAPI staining labels nuclei of both *P. brassicae* and host cells, whereas CFW binds to the β1–3 and β1–4 polysaccharides of cellulose and chitin, the latter is found in fungal cell walls and the cell walls of *P. brassicae* mature resting spores (Moxham & Buczacki, 1983; Schwelm et al., 2015). By using double‐staining with Nile red and DAPI, multiple small spherical primary plasmodia with abundant Nile red‐stained lipid droplets and a single punctated DAPI stain were observed in infected epidermal cells of *Arabidopsis* roots at the initial stages 24 hr post inoculation. Furthermore, double‐staining with Nile red and CFW identified, in real time, the deposition of cell wall chitin at the late stages of resting spore development. Taken together, confocal microscopy coupled with Nile red labeling and dual staining enabled the *in planta* observation of different forms of pathogen development including primary and secondary plasmodia and resting spores at different stages of infection, as well as their locations inside infected roots. To date, this was not possible to achieve in a timely manner or without extensive tissue damage.

Nile red staining and its compatibility with live cell imaging has been thoroughly studied in fungal, microalgae, animal and plant cells (Greenspan, Mayer, & Fowler, 1985; Guzmán et al., 2010; Kimura et al., 2004; Kopischke et al., 2013; Kucherak et al., 2010; Miquel et al., 2014; Rumin et al., 2015). In this study, simultaneous visualization of Nile red‐labeled *P. brassicae* structures and fluorescent protein tagging of *Arabidopsis* cellular components provided a novel approach capable of identifying dynamic cellular responses of host cells upon *P. brassicae* infection. Our results showed that primary plasmodia initially colonized at the cytoplasmic periphery of root epidermal cells. At this stage, *P. brassicae* infection seems to cause little disturbance to the host plant cell since infected cells displayed stable fluorescence signals when either the plasma membrane (PM) or the nucleus of *Arabidopsis* cells were labeled with a GFP fusion to a *Nicotiana plumbaginifolia* PM H^+^‐ATPase (PMA4‐GFP, Teh & Moore, 2007) or to a nuclear protein (AT4G19150‐GFP, Cutler et al, 2000), respectively. Later in the primary infection, primary plasmodia were identified in root epidermal cells showing a diminished PMA4‐GFP signal when compared to adjacent noninfected epidermal cells (Figure 6b). In a separate study using transgenic *Arabidopsis* plants expressing a γTIP‐GFP marker labeling the tonoplast, we observed that the γTIP‐GFP signals always disappeared in root epidermal cells with the primary plasmodia (unpublished data). These data suggest that the host cells harboring *P. brassicae* primary plasmodia might undergo a general degeneration process, or possible cell death, at this stage of the pathogen life cycle. Together, this live cell imaging provides new research opportunities for deciphering the temporal and spatial cellular responses involved in the biotrophic, intracellular *P. brassicae*–host interaction.

## CONFLICT OF INTEREST

The authors declare no conflict of interest.

## AUTHORS CONTRIBUTION

J.T. and J.B. performed experiments; J.T., P.B.S., and Y.W. conceived the project, analyzed the data, and wrote the paper with input from J.B.; P.B.S. and Y.W. designed the experiments.

## ETHICS STATEMENT

This study did not involve animal participants. Therefore, no ethics approval is required.

## Data Availability

All data are included in the manuscript.

## References

[mbo3765-bib-0001] Agarwal, A. , Kaul, V. , Faggian, R. , & Cahill, D. M. (2009). Development and use of a model system to monitor clubroot disease progression with an Australian field population of *Plasmodiophora brassicae* . Australasian Plant Pathology, 38(2), 120–127.

[mbo3765-bib-0002] Aist, J. R. , & Williams, P. H. (1971). The cytology and kinetics of cabbage root hair penetration by *Plasmodiophora brassicae* Wor. Canadian Journal of Botany, 49, 2023–2034. 10.1139/b71-284

[mbo3765-bib-0003] Asano, T. , & Kageyama, K. (2006). Growth and movement of secondary plasmodia of *Plasmodiophora brassicae* in turnip suspension‐culture cells. Plant Pathology, 55(1), 145–151. 10.1111/j.1365-3059.2006.01320.x

[mbo3765-bib-0004] Asano, T. , Kageyama, K. , & Hyakumachi, M. (2000). Germination of surface disinfected resting spores of *Plasmodiophora brassicae* and their root hair infection in turnip hairy roots. Mycoscience, 41(11), 49–54. 10.1007/BF02464385

[mbo3765-bib-0005] Asano, T. , Kodama, A. , & Kageyama, K. (2006). Susceptibility of hairy root lines of *Brassica* species to *Plasmodiophora brassicae* and in an in vitro subculture system. Journal of General Plant Pathology, 72(2), 85–91. 10.1007/s10327-005-0253-9

[mbo3765-bib-0006] Bulman, S. , Candy, J. M. , Fiers, M. , Lister, R. , Conner, A. J. , & Eady, C. C. (2011). Genomics of biotrophic, plant‐infecting plasmodiophorids using in vitro dual culture. Protist, 162(3), 449–461. 10.1016/j.protis.2010.09.004 21183405

[mbo3765-bib-0007] Cutler, S. R. , Ehrhardt, D. W. , Griffitts, J. S. , & Somerville, C. R. (2000). Random GFP:cDNA fusions enable visualization of subcellular structures in cells of Arabidopsis at a high frequency. Proceeding of the National Academy of Sciences of the United States of American, 97(7), 3718–3723. 10.1073/pnas.97.7.3718 PMC16306 10737809

[mbo3765-bib-0008] Deora, A. , Gossen, B. D. , & McDonald, M. R. (2013). Cytology of infection, development and expression of resistance to *Plasmodiophora brassicae* in canola. Annals of Applied Biology, 163, 56–71. 10.1111/aab.12033

[mbo3765-bib-0009] Diaz, G. , Melis, M. , Batetta, B. , Angius, F. , & Falchi, A. M. (2008). Hydrophobic characterization of intracellular lipids in situ by Nile Red red/yellow emission ratio. Micron, 39(7), 819–824. 10.1016/j.micron.2008.01.001 18329888

[mbo3765-bib-0010] Dixon, G. R. (2009). The occurrence and economic impact of *Plasmodiophora brassicae* and clubroot disease. Journal of Plant Growth Regulation, 28(3), 194–202. 10.1007/s00344-009-9090-y

[mbo3765-bib-0011] Donald, E. C. , & Porter, I. J. (2004). A sand‐solution culture technique used to observe the effect of calcium and pH on root hair and cortical stages of infection by *Plasmodiophora brassicae* . Australasian Plant Pathology, 33(4), 585–589.

[mbo3765-bib-0012] Fei, W. , Feng, J. , Rong, S. , Strelkov, S. E. , Gao, Z. , & Hwang, S.‐F. (2016). Infection and gene expression of the clubroot pathogen *Plasmodiophora brassicae* in resistant and susceptible canola cultivars. Plant Disease, 100(4), 824–828. 10.1094/pdis-11-15-1255-re 30688612

[mbo3765-bib-0013] Greenspan, P. , Mayer, E. P. , & Fowler, S. D. (1985). Nile red: A selective fluorescent stain for intracellular lipid droplets. Jornal of Cell Biology, 100(3), 965–973. 10.1083/jcb.100.3.965 PMC2113505 3972906

[mbo3765-bib-0014] Guzmán, H. M. , De la Jara, A. , Duarte, L. C. , & Presmanes, K. F. (2010). Estimate by means of flow cytometry of variation in composition of fatty acids from *Tetraselmis suecica* in response to culture conditions. Aquaculture International, 18(2), 189–199. 10.1007/s10499-008-9235-1

[mbo3765-bib-0015] Hwang, S.‐F. , Ahmed, H. U. , Zhou, Q. , Strelkov, S. E. , Gossen, B. D. , Peng, G. , & Turnbull, G. D. (2011). Influence of cultivar resistance and inoculum density on root hair infection of canola (*Brassica napus*) by *Plasmodiophora brassicae* . Plant Pathology, 60, 820–829. 10.1111/j.1365-3059.2011.02457.x

[mbo3765-bib-0016] Kageyama, K. , & Asano, T. (2009). Life cycle of *Plasmodiophora brassicae* . Journal of Plant Growth Regulation, 28, 203 10.1007/s00344-009-9101-z

[mbo3765-bib-0017] Kimura, K. , Yamaoka, M. , & Kamisaka, Y. (2004). Rapid estimation of lipids in oleaginous fungi and yeasts using Nile red fluorescence. Journal of Microbiology Methods, 56(3), 331–338. 10.1016/j.mimet.2003.10.018 14967224

[mbo3765-bib-0018] Kopischke, M. , Westphal, L. , Schneeberger, K. , Clark, R. , Ossowski, S. , Wewer, V. , … Rosahl, S. (2013). Impaired sterol ester synthesis alters the response of *Arabidopsis thaliana* to *Phytophthora infestans* . Plant Journal, 73(3), 456–468. 10.1111/tpj.12046 23072470

[mbo3765-bib-0019] Kucherak, O. A. , Oncul, S. , Darwich, Z. , Yushchenko, D. A. , Arntz, Y. , Didier, P. , … Klymchenko, A. S. (2010). Switchable Nile red‐based probe for cholesterol and lipid order at the outer leaflet of biomembranes. Journal of the American Chemical Society, 132(13), 4907–4916. 10.1021/ja100351w 20225874

[mbo3765-bib-0020] Li, R. , Sun, R. , Hicks, G. R. , & Raikhel, N. V. (2015). *Arabidopsis* ribosomal proteins control vacuole trafficking and developmental programs through the regulation of lipid metabolism. Proceeding of the National Academy of Sciences of the United States of American, 112(1), E89–E98. 10.1073/pnas.1422656112 PMC4291620 25535344

[mbo3765-bib-0021] Miquel, M. , Trigui, G. , d'Andréa, S. , Kelemen, Z. , Baud, S. , Berger, A. , … Dubreucq, B. (2014). Specialization of oleosins in oil body dynamics during seed development in Arabidopsis seeds. Plant Physiology, 164(4), 1866–1878. 10.1104/pp.113.233262 24515832 PMC3982749

[mbo3765-bib-0022] Moxham, S. , & Buczacki, S. T. (1983). Chemical composition of the resting spore well of *Plasmodiophora brassicae* . Transactions of the British Mycological Society, 80(2), 297–304. 10.1016/s0007-1536(83)80013-8

[mbo3765-bib-0023] Peng, G. , McGregro, L. , Lahlali, R. , Gosson, B. D. , Hwang, S.‐H. , Adhikar, K. K. , … McDonald, M. R. (2011). Potential biological control of clubroot on canola and crucifer vegetable crops. Plant Pathology, 60, 566–574. 10.1111/j.1365-3059.2010.02400.x

[mbo3765-bib-0024] Rumin, J. , Bonnefond, H. , Saint‐Jean, B. , Rouxel, C. , Sciandra, A. , Bernard, O. , … Bougaran, G. (2015). The use of fluorescent Nile red and BODIPY for lipid measurement in microalgae. Biotechnology for Biofuels, 8, 42 10.1186/s13068-015-0220-4 25788982 PMC4364489

[mbo3765-bib-0025] Schuller, A. , & Ludwig‐Müller, J. (2016). Histological methods to detect the clubroot pathogen *Plasmodiophora brassicae* during its complex life cycle. Plant Pathology, 65, 1223–1237. 10.1111/ppa.12520

[mbo3765-bib-0026] Schwelm, A. , Fogelqvist, J. , Knaust, A. , Jülke, S. , Lilja, T. , Bonilla‐Rosso, G. , … Dixelius, C. (2015). The *Plasmodiophora brassicae* genome reveals insights in its life cycle and ancestry of chitin synthases. Scientific Report, 5, 11153 10.1038/srep11153 PMC4471660 26084520

[mbo3765-bib-0027] Sharma, K. , Gossen, B. D. , & McDonald, M. R. (2011a). Effect of temperature on cortical infection by *Plasmodiophora brassicae* and clubroot severity. Phytopathology, 101(12), 1424–1432. 10.1094/phyto-04-11-0124 21864086

[mbo3765-bib-0028] Sharma, K. , Gossen, B. D. , & Mcdonald, M. R. (2011b). Effect of temperature on primary infection by *Plasmodiophora brassicae* and initiation of clubroot symptoms. Plant Pathology, 60, 830–838. 10.1111/j.1365-3059.2011.02458.x

[mbo3765-bib-0029] Siemens, J. , Nagel, M. , Ludwig‐Müller, J. , & Sacristán, M. D. (2002). The interaction of *Plasmodiophora brassicae* and *Arabidopsis thaliana*: Parameters for disease quantification and screening of mutant lines. Journal of Phytopathology, 150, 592–605. 10.1046/j.1439-0434.2002.00818.x

[mbo3765-bib-0030] Takahashi, H. , Ishikawa, T. , Kaido, M. , Takita, K. , Tayakawa, T. , Okazaki, K. , … Hori, H. (2006). *Plasmodiophora brassicae*‐induced cell death and medium alkalization in clubroot‐resistant cultured roots of *Brassica rapa* . Journal of Phytopathology, 154, 156–162. 10.1111/j.1439-0434.2006.01076.x

[mbo3765-bib-0031] Takahashi, H. , Muraoka, S. , Ito, K. , Mitsui, T. , Hori, H. , & Kiso, A. (2001). Resting spores of *Plasmodiophora brassicae* proliferation only in the callus of clubroot disease‐susceptible turnip but increases PAL activity in the callus of clubroot disease‐resistant turnip. Plant Biotechnology, 18(4), 267–274. 10.5511/plantbiotechnology.18.267

[mbo3765-bib-0032] Teh, O. K. , & Moore, I. (2007). An ARF‐GEF acting at the Golgi and in selective endocytosis in polarized plant cells. Nature, 448, 493–496. 10.1038/nature06023 17653191

[mbo3765-bib-0033] Verbelen, J. P. , De Cnodder, T. , Le, J. , Vissenberg, K. , & Baluška, F. (2006). The root apex of *Arabidopsis thaliana* consists of four distinct zones of growth activities. Plant Signal Behavior, 1(6), 296–304. 10.4161/psb.1.6.3511 PMC2634244 19517000

[mbo3765-bib-0034] Williams, P. H. , & McNabola, S. (1967). Fine structure of *Plasmodiophora brassicae* in sporogenesis. Canadian Journal of Botany, 45, 1165–1169. 10.1139/b67-173

[mbo3765-bib-0035] Williams, P. H. , & Yukawa, Y. B. (1967). Ultrastructure studies on the host parasite relations of *Plasmodiophora brassicae* . Phytopathology, 57, 682.

